# Hypoxia Modifies the Transcriptome of Human NK Cells, Modulates Their Immunoregulatory Profile, and Influences NK Cell Subset Migration

**DOI:** 10.3389/fimmu.2018.02358

**Published:** 2018-10-16

**Authors:** Monica Parodi, Federica Raggi, Davide Cangelosi, Claudia Manzini, Mirna Balsamo, Fabiola Blengio, Alessandra Eva, Luigi Varesio, Gabriella Pietra, Lorenzo Moretta, Maria Cristina Mingari, Massimo Vitale, Maria Carla Bosco

**Affiliations:** ^1^UOC Immunologia, IRCCS Ospedale Policlinico San Martino, Genova, Italy; ^2^Laboratorio di Biologia Molecolare, IRCCS Istituto Giannina Gaslini, Genova, Italy; ^3^Laboratorio di Immunologia Clinica e Sperimentale, IRCCS Istituto Giannina Gaslini, Genova, Italy; ^4^Dipartimento di Medicina Sperimentale, Università di Genova, Genova, Italy; ^5^Immunology Area, Ospedale Pediatrico Bambin Gesù, Rome, Italy; ^6^Center of Excellence for Biomedical Research, University of Genoa, Genova, Italy

**Keywords:** NK cells, hypoxia, tumor immunology, cytokines/chemokines, chemokine receptors, CD56bright cells, tumor infiltration, transcriptome

## Abstract

Hypoxia, which characterizes most tumor tissues, can alter the function of different immune cell types, favoring tumor escape mechanisms. In this study, we show that hypoxia profoundly acts on NK cells by influencing their transcriptome, affecting their immunoregulatory functions, and changing the chemotactic responses of different NK cell subsets. Exposure of human peripheral blood NK cells to hypoxia for 16 or 96 h caused significant changes in the expression of 729 or 1,100 genes, respectively. Gene Set Enrichment Analysis demonstrated that these changes followed a consensus hypoxia transcriptional profile. As assessed by Gene Ontology annotation, hypoxia-targeted genes were implicated in several biological processes: metabolism, cell cycle, differentiation, apoptosis, cell stress, and cytoskeleton organization. The hypoxic transcriptome also showed changes in genes with immunological relevance including those coding for proinflammatory cytokines, chemokines, and chemokine-receptors. Quantitative RT-PCR analysis confirmed the modulation of several immune-related genes, prompting further immunophenotypic and functional studies. Multiplex ELISA demonstrated that hypoxia could variably reduce NK cell ability to release IFNγ, TNFα, GM-CSF, CCL3, and CCL5 following PMA+Ionomycin or IL15+IL18 stimulation, while it poorly affected the response to IL12+IL18. Cytofluorimetric analysis showed that hypoxia could influence NK chemokine receptor pattern by sustaining the expression of CCR7 and CXCR4. Remarkably, this effect occurred selectively (CCR7) or preferentially (CXCR4) on CD56^bright^ NK cells, which indeed showed higher chemotaxis to CCL19, CCL21, or CXCL12. Collectively, our data suggest that the hypoxic environment may profoundly influence the nature of the NK cell infiltrate and its effects on immune-mediated responses within tumor tissues.

## Introduction

NK cells are powerful effectors of the innate immunity with anti-tumor activity (1–5). They are endowed with a unique pattern of receptors sensing changes in MHC-I expression levels (which are often decreased in tumor cells) or recognizing ligands induced by tumor transformation, cell stress, and DNA damage (1, 5–8). By these receptors NK cells can direct their potent lytic machinery to target and eliminate many tumor cell types (6). In addition, NK cells can release pro-inflammatory cytokines and various chemotactic factors (IFNγ, TNFα, GM-CSF, CCL3/CCL4) potentially amplifying immune responses to the tumor (1, 6, 9–11). The cytolytic function and the capability of releasing cytokines and chemokines appear to be differently represented in the two major subsets of peripheral blood (PB)-NK cells, characterized by the CD56^dim^/CD16^bright^ (CD56^dim^) or the CD56^bright^/CD16^dim/neg^ (CD56^bright^) phenotype (1, 12–14). The CD56^dim^ cells are strongly cytotoxic and can also produce cytokines in response to specific stimuli. These cells represent the large majority of the PB-NK cell population and express chemokine receptors, mainly CXCR1 and CX_3_CR1, that enable their recruitment to inflamed tissues (1, 9, 11, 15, 16). Conversely, CD56^bright^ cells are poorly cytotoxic and release high amount of cytokines, especially in response to monokines. According to their expression of CCR7 and CD62L, these cells are mainly located within secondary lymphoid compartments while they account for only 10% of PB-NK cells (1, 9, 11, 15).

The increasing interest on NK cells as potential tools for immunotherapy has recently inspired many studies aimed at defining how their anti-tumor activity can be influenced by the tumor microenvironment. Along this line, different suppressive interactions mediated by tumor cells, tumor-associated fibroblasts, or regulatory immune cells have been described and characterized (17–22). In addition, it has been shown that tumor cells can escape NK cell attack by modulating the surface expression of various NK-receptor ligands (2, 23–27). In spite of these important advances in the field, a crucial issue that still remains to be investigated for an effective exploitation of NK cells in the therapy of solid tumors is the recruitment of NK cells to tumor tissues. Few recent studies have shown that higher NK cell infiltration correlates with a better prognosis of the disease (23, 28, 29), but have also indicated that the NK cell infiltrate in tumor tissues is often poor and, in some cases, mostly represented by poorly cytotoxic CD56^bright^ cells (5, 30, 31). Specific chemokine milieus, or alteration of chemokine receptor patterns, may account for these findings. However, an exhaustive explanation on how the tumor microenvironment can influence NK cell infiltration has not yet been achieved.

Reduced partial O_2_ tension (pO_2_, 0–20 mm Hg, hypoxia), which often affects tumor tissues, may play role in this context. Hypoxia is an important driver of malignant progression and resistance to therapy (23, 32, 33). It can influence the function of different cell types within the tumor lesion and affect the recruitment of immune cells, favoring tumor escape mechanisms (33). Indeed, exposure to hypoxia can induce different immune and non-immune cells to change the expression of pro-angiogenetic factors, cytokines, and chemokines (including VEGF, SPP1, IL-1β, MIF, CXCL12, and CXCL8), or chemokine receptors (including CXCR4, CCR2, and CCR5) (34–38). In spite of many studies on this issue, limited information is currently available on the impact of hypoxia on NK cells and their subsets, notably on their ability to respond to specific chemotactic stimuli or to release immune-active soluble factors (39–41). We have previously shown that, in NK cells exposed to IL-2, hypoxia can down-regulate expression and function of most NK cell receptors that activate cytolytic activity against tumor or virally infected cells, but preserves NK cell ability to kill targets via ADCC (39), suggesting that NK cells may be effective even in hypoxic niches in the context of combined immunotherapeutic approaches. In this study, we integrate previous data and provide an overview of the effect of hypoxia on NK cells stimulated with IL-2. Moreover, we indicate some clues on how the composition and the function of NK cell infiltrate may be influenced by the hypoxic environment in tumor tissues.

## Materials and methods

### NK cells isolation and culture

NK cells were obtained from PB of healthy donors provided by the transfusion center of the Ospedale Policlinico San Martino following approved internal operational procedures (IOH78). Written informed consent from the donors was provided according to the Declaration of Helsinki. Briefly: NK cells from healthy donors were isolated from PB mononuclear cells using RosetteSep NK Cell Enrichment Cocktail (StemCell Technologies, 15025 Vancouver, Canada). Only preparations displaying more than 95% of CD56+ CD3– CD14– NK cells were selected for the experiments. After isolation, NK cells were cultured for the indicated time points in RPMI 1640 (Lonza Verviers, Belgium) supplemented with 10% Fetal Bovine Serum (FBS, Voden Medical S.p.a. Meda MB, Italy), antibiotic mixture (0.05 mg/mL penicillin, 0.05 mg/mL streptomycin Lonza, Verviers, Belgium), and 100 U/mL recombinant human IL-2 (Proleukin, Novartis Basilea, Switzerland) at 2X 10^6^cells/mL in round bottom 96-well microtiter plates. The cultures were performed either under normoxic conditions in a humidified incubator containing 20% O_2_, 5% CO_2_, and 75% N_2_ or under hypoxic conditions. Hypoxic conditions were obtained by culturing cells in a sealed anaerobic workstation incubator (Ruskinn, INVIVO_2_ 400, CARLI Biotec, Roma, Italy), incorporating a gas mixing system (Ruskinn Gas Mixer Q) and flushed with a mixture of 1% O_2_, 5% CO_2_, and 94% N_2_.

### RNA isolation and cRNA synthesis

Total RNA was purified from NK cells derived from three healthy donors using the RNeasy MiniKit from Qiagen (Milano, Italy). RNA was controlled for integrity by nanoelectrophoresis with an Agilent 2100 Bioanalyzer (Agilent Technologies Europe, Waldbroon, Germany), quantified by spectrophotometry using a NanoDrop ND-1000 (NanoDrop Technologies, Wilmington, USA), and reverse-transcribed into double-stranded cDNA on a GeneAmp PCR System 2700 thermal cycler (Applied Biosystems, Milano) using the one-cycle cDNA synthesis kit (Affymetrix, Milano). cDNA derived from three donors/time point was purified and biotin labeled using the GeneChip IVT kit (Affymetrix). Labeled cRNA was fragmented according to Affymetrix's instructions.

### GeneChip hybridization and array data analysis

Gene expression profiling was performed as described previously (42). Briefly, Fragmented cRNA was hybridized on the Affymetrix HG-U133 plus 2.0 GeneChips (Genopolis Corporation, Milano) containing 54,000 probe sets (coding for 47,000 transcripts and variants, including 38,500 unique human genes) on a single array. Chips were stained with streptavidin-phycoerythrin (Invitrogen Life Technologies, Milano) and scanned using an Affymetrix GeneChip Scanner 3000, as described. Data were processed by RMA normalization utilizing the “Affy” R package. Statistical analysis using paired *t*-test was performed to identify differentially expressed genes. We corrected the *p*-value for multiple hypothesis testing by Benjamini–Hochberg method to false discovery rate control. Only gene differences that passed the test at a confidence level of 95% (*P* < 0.05) and a false discovery rate of 0.05% were considered significant. Fold-change (FC) was calculated as the ratio between the average expression level under hypoxia and normoxia. Genes were defined as being differentially regulated by hypoxia if they exhibited more than 2-fold increase in gene expression or down-regulated if they showed <0.5-fold change compared with normoxic cultures. We converted the Affymetrix probe sets into the corresponding gene symbol by Netaffx tool. When multiple probe sets were associated with the same gene symbol, the probe set with the highest expression signal was considered. The full set of data from each microarray experiment has been deposited in the Gene Expression Omnibus public repository at NCBI (www.ncbi.nlm.nih.gov) and is accessible through GEO (Accession number GSE116660). Biological processes were assessed by DAVID Gene Ontology (GO) enrichment analysis (http://david.niaid.nih.gov). The significant GO terms were defined as *p* < 0.05 and FDR < 0.05.

### Gene set enrichment analysis

Gene Set Enrichment Analysis (GSEA) was performed on all probe sets of the Affymetrix HG-U133 Plus 2.0 GeneChip, as described previously (43). An enrichment score (ES) and a normalized enrichment score (NES) were calculated for every gene set. The statistical significance of NES was estimated by an empirical test using 1,000 gene set permutations to obtain the nominal *p*-value. A false discovery rate (FDR) q value was estimated to control the probability that a NES could represent a false positive finding. The gene sets used in the GSEA belong to the C2.CGP collection of the Broad Institute Molecular Signature v5 Database (MSigDB) (44). The analysis used Signal-to-Noise metric and considered gene sets containing at least 15 and up to 250 probe sets. An enrichment with FDR q-values lower than 0.05 and nominal *p* < 0.05 was considered significant. Leading Edge Analysis (LEA) of enriched gene sets was used to identify key genes related to NK response to hypoxia.

### Real-time RT-PCR

cDNA was prepared from purified total RNA using SuperScript Double-Stranded cDNA synthesis kit (Invitrogen). Real time PCR (qRT-PCR) was performed on a 7500 Real Time PCR System (Applied) in triplicate for each target transcript using SYBR Green PCR Master Mix and sense/antisense oligonucleotide primers synthesized by TIBMolbiol (Genova) or purchased from Quiagen, as detailed before (45). Expression data were normalized on the values obtained in parallel for three reference genes (actin related protein 2/3 complex subunit 1B, ARCP1B; ribosomal proteins S18, RSP18; and RSP19), using the Bestkeeper software, and relative expression values were calculated using Q-gene software, as detailed (45).

### mAbs and flow cytofluorimetric analysis

The following mAbs were used in this study: anti-CCR1 (R&D System, MAB 145-100, Minneapolis U.S.A.), anti-CCR5 (R&D System, MAB 182-100 Minneapolis U.S.A.), anti-CCR7 (R&D System, MAB 197-100 Minneapolis U.S.A.), anti-CXCR1/IL-8 RA (R&D System, MAB 173-100 Minneapolis U.S.A.), anti-CXCR3 (R&D System, MAB 160-100), anti-CXCR4 (R&D System, MAB 173-100), PE-conjugated anti-CX_3_CR1 (Medical & Biological Laboratories Co., LTD, D070-5), FITC-conjugated anti-CD3 (eBioscience, 11-0038-42 Thermofisher scientific, Waltham, Massachusetts, Stati Uniti), PE-cyanine 7-conjugated anti-CD56 (Beckman Coulter, A21692, Brea, California U.S.A.), PE-conjugated anti-CD16 (130-106-704, Miltenyi Biotec Bergisch Gladbach, Germany). The staining with the appropriate unlabeled mAbs are followed by PE-conjugated isotype-specific goat anti-mouse second reagent (Southern Biotechnology Associated, Birmingham, AL, U.S.A.), and fluorescence was quantified on a Gallios™ Flow Cytometer (Beckman Coulter, Brea, California U.S.A.).

### Multiplex ELISA analyses

Freshly isolated NK cells were cultured for 20 h at 5X 10^5^/mL in flat bottom 96-well microtiter plates in the presence of the following recombinant human cytokines: IL-2, IL-12+IL-18, or IL-15+IL-18. The cytokine concentrations were: 100 U/mL IL-2 (Proleukin, Novartis Basilea, Switzerland); 2.5 ng/mL IL-12 (Peprotech, 200-12 London, UK); 20 ng/mL IL-15 (Peprotech, 200-15 London, UK); 200 ng/mL IL-18 (Medical & Biological Laboratories Co. LTD, B001-5, Japan). In the “PMA+IONO IL-2” condition, NK cells were cultured in the presence of IL-2 for 26 h, and 100 ng/mL PMA (Phorbol 12-myristate 13 acetate, SIGMA-Aldrich Saint Louis, Missouri, U.S.A.) and 500 ng/mL IONO (Ionomycin, SIGMA-Aldrich, Missouri, U.S.A.) were added to the cultures for the last 6 h. The cultures were performed in parallel under normoxic and hypoxic conditions (see above). Culture supernatants were then collected and analyzed for their cytokine content by MAGPIX® System (Luminex® xMAP® Technology, Merck Millipore, Germany).

### Chemotaxis assay

NK cells freshly isolated from peripheral blood and then cultured for different time points (24, 48, and 96 h) with IL-2 under hypoxic or normoxic conditions were seeded at 2.5 × 10^6^/mL in the upper chamber of a Transwell system (3 mm pore size; Corning Costar, 3415). 10% FBS RPMI 1640 medium alone or supplemented with recombinant human CXCL12 [100 ng/mL] (Peprotech, 300-28A), or CCL19 [0.3 μg/mL], or CCL21 [0.6 μg/mL] was added to the lower compartment. Cells were allowed to migrate for 2 h at 37°C under normoxic condition. Cells migrated in the lower chamber were collected and counted using the MACSQuant Analyzer (Miltenyi Biotec Bergisch Gladbach, Germany) or analyzed with the Gallios™ Flow Cytometer after surface double staining of CD56/CD16 markers. Cells migrated in the lower chamber containing medium alone (w/o chemokines) represented spontaneous migration due to unspecific cell motility. Chemotactic response was assessed as percentage of spontaneous migration and was calculated as follows: (number of migrated cells in the presence of chemotactic stimulus/number of migrated cells in the absence of stimulus) × 100. The chemotactic response of CD56^bright^CD16^dim/neg^ NK cells to CCL19, CCL21, and CXCL12 was assessed as enrichment of this specific cell subset within migrated cells and was calculated as follows: CD56^bright^CD16^dim/neg^ cell percentage within cells migrated in response to chemokines/CD56^bright^CD16^dim/neg^ cell percentage within spontaneously migrated cells.

### Statistical analysis

Statistical analyses were performed using the Prism software package (GraphPad Software). Data are expressed as the mean ± SEM of at least three independent experiments, unless differently specified. Statistical significance was evaluated by two-tailed paired Student's *t-*test. A *p* < 0.05 (^*^), < 0.01(^**^), or < 0.001(^***^) was considered statistically significant.

## Results

### Gene expression profile of hypoxic NK cells

**Figure 1:**
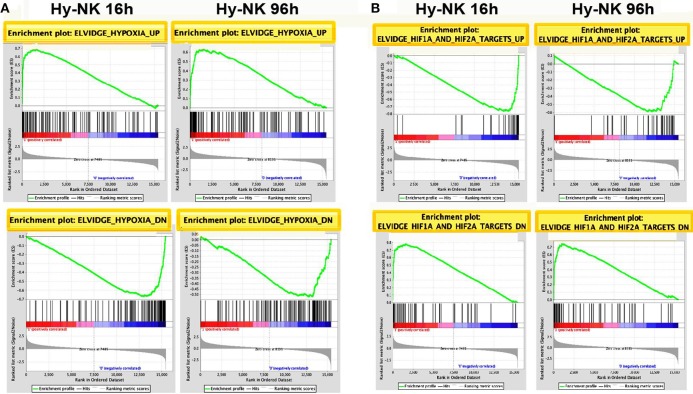
Gene Set Enrichment Analysis (GSEA) Plots for representative hypoxia- or HIF1α/2α-related gene sets in Hy-NK cell transcriptomes. The transcripts identified by microarray analysis in NK cells were ranked by level of hypoxia-mediated up- or down-regulation. The ranked gene lists were then compared by GSEA with previously published gene sets for hypoxia-regulated genes or for genes previously shown to be HIF targets in other cell types. **(A)** GSEA plots of representative sets of up- or down-regulated genes from cells exposed to hypoxia (ELVIDGE_HYPOXIA_UP or _DN, respectively). **(B)** GSEA plots of representative sets of up- or down-regulated genes from cells undergoing HIF-1α and HIF-2α silencing (Elvidge_HIF1A_and_HIF2A_TARGETS_UP or _DN, respectively). Note that in the case HIF1α/2α-silencing the sets of up- or down-regulated genes resulted inversely enriched in the Hy-NK cell transcriptomes. The enrichment score is calculated by walking down a list of genes ranked by their correlation with the phenotype, increasing a running-sum statistic when a gene in that gene set is encountered (each black vertical line underneath the enrichment plot) and decreasing it when a gene that isn't in the gene set is encountered. The enrichment score is the maximum deviation from zero encountered in the walk.

To obtain an overview of NK cell response to hypoxia, we assessed the gene expression profile of NK cells isolated from the PB of three independent healthy donors and cultured with IL-2 for 16 or 96 h under hypoxic (1% O_2_) or normoxic (20% O_2_) conditions. mRNA was individually hybridized to human Affymetrix HG-U133 plus 2.0 GeneChips, obtaining three biological replicates for each experimental condition. Raw data were processed as described in the section Materials and Methods. We used GSEA to determine the enrichment of the published C2.CGP gene set collection (44) in the 16 and 96 h transcriptomes of hypoxic NK cells (Hy-NK) as compared to their normoxic counterparts. We selected 25 gene sets using “hypoxia” and “hypoxia-inducible factor (HIF)” as keywords (see section Materials and Methods for details). The list of gene sets, their normalized enrichment score (NES), false discovery rate *q*-values (FDR q-val), and nominal *p*-values (NOM p-val) are reported in Table 1. Among selected gene sets, 20 were significantly enriched in both Hy-NK cell transcriptomes (*p* < 0.05; FDR *q* < 0.05), 1 and 3 additional gene sets were specifically enriched in the 16 h (upregulated) and the 96 h (downregulated) hypoxic transcriptomes, respectively, and only 1 gene set (upregulated) was not significantly enriched at either time points. Representative 16 and 96 h plots showing clear enrichment of the gene sets at the top or the bottom of the ranked list are presented in Figure 1A for a visual inspection of the GSEA results (46). These data demonstrate that gene expression changes in Hy-NK cells follow a consensus hypoxia transcriptional profile.

**Table 1:** Hypoxia- and HIF-related gene sets enriched in the 16 and 96 h hy-NK cell transcriptomes.

**GSEA term^a^**	**16 h up**				**96 h up**			
	**Size^b^**	**NES^c^**	**FDR *q*-val^d^**	**Nom *p*-val^e^**	**Size**	**NES**	**FDR *q-*val**	**Nom *p*-val**
ELVIDGE_HIF1A_TARGETS_DN	69	3.26	<0.001	<0.001	69	2.98	<0.001	<0.001
ELVIDGE_HIF1A_AND_HIF2A_TARGETS_DN	75	3.17	<0.001	<0.001	75	2.96	<0.001	<0.001
MENSE_HYPOXIA_UP	75	3.16	<0.001	<0.001	75	2.70	<0.001	<0.001
ELVIDGE_HYPOXIA_UP	130	3.06	<0.001	<0.001	130	2.78	<0.001	<0.001
ELVIDGE_HYPOXIA_BY_DMOG_UP	102	3.03	<0.001	<0.001	102	2.85	<0.001	<0.001
FARDIN_HYPOXIA_11	25	2.92	<0.001	<0.001	25	2.75	<0.001	<0.001
SEMENZA_HIF1_TARGETS	28	2.70	<0.001	<0.001	28	2.09	0.003	<0.001
LEONARD_HYPOXIA	33	2.67	<0.001	<0.001	33	2.55	<0.001	<0.001
KIM_HYPOXIA	18	2.62	<0.001	<0.001	18	2.02	0.005	<0.001
HARRIS_HYPOXIA	63	2.60	<0.001	<0.001	63	1.91	0.018	<0.001
GROSS_HIF1A_TARGETS_DN	17	2.59	<0.001	<0.001	17	1.75	0.064	0.006
WINTER_HYPOXIA_METAGENE	184	2.55	<0.001	<0.001	184	2.21	<0.001	<0.001
QI_HYPOXIA	107	2.47	<0.001	<0.001	107	2.01	0.005	<0.001
MANALO_HYPOXIA_UP	163	2.34	<0.001	<0.001	163	2.63	<0.001	<0.001
GROSS_HYPOXIA_VIA_ELK3_AND_HIF1A_UP	108	2.15	0.001	<0.001	108	1.96	0.01	<0.001
GROSS_HYPOXIA_VIA_ELK3_DN	125	1.52	0.1934	0.000	125	1.18	0.5	1
**GSEA term**	**16h down**				**96h down**			
	**Size**	**NES**	**FDR** ***q*****-val**	**Nom** ***p*****-val**	**Size**	**NES**	**FDR** ***q*****-val**	**Nom** ***p*****-val**
MANALO_HYPOXIA_DN	236	−3.20	<0.001	<0.001	236	−3.34	<0.001	<0.001
ELVIDGE_HYPOXIA_DN	123	−2.84	<0.001	<0.001	123	−2.14	<0.001	<0.001
ELVIDGE_HIF1A_TARGETS_UP	56	−2.71	<0.001	<0.001	56	−1.88	0.003	<0.001
ELVIDGE_HIF1A_AND_HIF2A_TARGETS_UP	37	−2.62	<0.001	<0.001	37	−2.00	<0.001	<0.001
ELVIDGE_HYPOXIA_BY_DMOG_DN	49	−2.42	<0.001	<0.001	49	−2.12	<0.001	<0.001
GROSS_HYPOXIA_VIA_HIF1A_UP	60	−2.11	<0.001	<0.001	60	−1.81	0.007	<0.001
GROSS_HYPOXIA_VIA_ELK3_AND_HIF1A_DN	77	−1.57	0.0678	0.0051	77	−1.87	0.003	<0.001
GROSS_HYPOXIA_VIA_ELK3_UP	166	−1.43	0.1474	0.0118	166	−2.28	<0.001	<0.001
GROSS_HYPOXIA_VIA_ELK3_ONLY_DN					37	−1.85	0.004	<0.001

*Microarray analysis was carried out on NK cells from 3 different donors cultured under normoxic (20% O2) and hypoxic (1% O2) conditions for 16 and 96 h. Differentially expressed transcripts were ranked by level of hypoxia-mediated up- or down-regulation. The ranked gene lists were then compared with published gene sets for hypoxia-regulated genes or for genes previously shown to be HIF targets in other cell types by GSEA*.

a *Gene sets enriched in the GSEA analysis. Gene sets belonged to the C2.CGP collection of the MSigDB and were selected using the keywords “hypoxia” and “HIF” and filtering out those having <15 probe sets and more than 250 probe sets. “Up” indicates genes enriched in the hypoxia transcriptomes (i.e., up-regulated in hypoxic NK cells); ”down" indicates genes enriched in the normoxia transcriptomes (i.e., down-regulated in hypoxic NK cells)*.

b *Relative number of probe sets in the gene sets*.

c *Normalized enrichment score of the gene sets. Gene sets are listed in decreasing order of NES*.

d *FDR q-value of the false discovery rate. Values ≤ 0.05 are considered acceptable*.

e *NOM p-value of the normalized enrichment score. Values ≤ 0.05 are considered significant*.

Gene transcriptional activation by hypoxia is mediated primarily by HIF, a heterodimer of a constitutive HIF-1β subunit and an O_2_-sensitive α-subunit (HIF-1α or HIF-2α) (32, 34). Interestingly, some of the selected gene sets were from cells undergoing HIF-1α or HIF-2α silencing (46). The reported sets of down- or up-regulated genes were found inversely enriched in the Hy-NK cell transcriptomes (Table 1—gene sets 1, 2, 19, 20—and Figure 1B), suggesting that HIF-1α and HIF-2α and their target genes could play an important role in NK cell response to hypoxia.

Leading Edge Analysis (LEA) applied to the significantly enriched gene sets allowed to define the subsets of hypoxia-related genes with the highest impact on the enrichment score (referred to as the leading edge subset) at 16 or 96 h (Figure S1). These subsets include genes involved in glycolysis, gluconeogenesis, and glucose transport (ALDOA, ALDOC, ENO1, ENO2, GAPDH, GPI, HK1, HK2, LDHA, PDK1, PGK1, SLC2A1, TPI1), non-glycolytic metabolism and ion transport (P4HA1, P4HA2, PAM, VLDLR), apoptosis, stress response, and proliferation (BNIP3, BNIP3L, CCNG2, DDIT3, EGLN1, EGLN3, NDRG1), transcription and signaling activity (FOSL2, JAK2, JUN, MXI1, SOCS2). These results extend to Hy-NK cells the expression of a large cluster of hypoxia-related genes previously identified in other cell types, including tumor and immune cells (32, 34, 47–49).

### Functional assessment of genes modulated by hypoxia in NK cells

**Figure 2:**
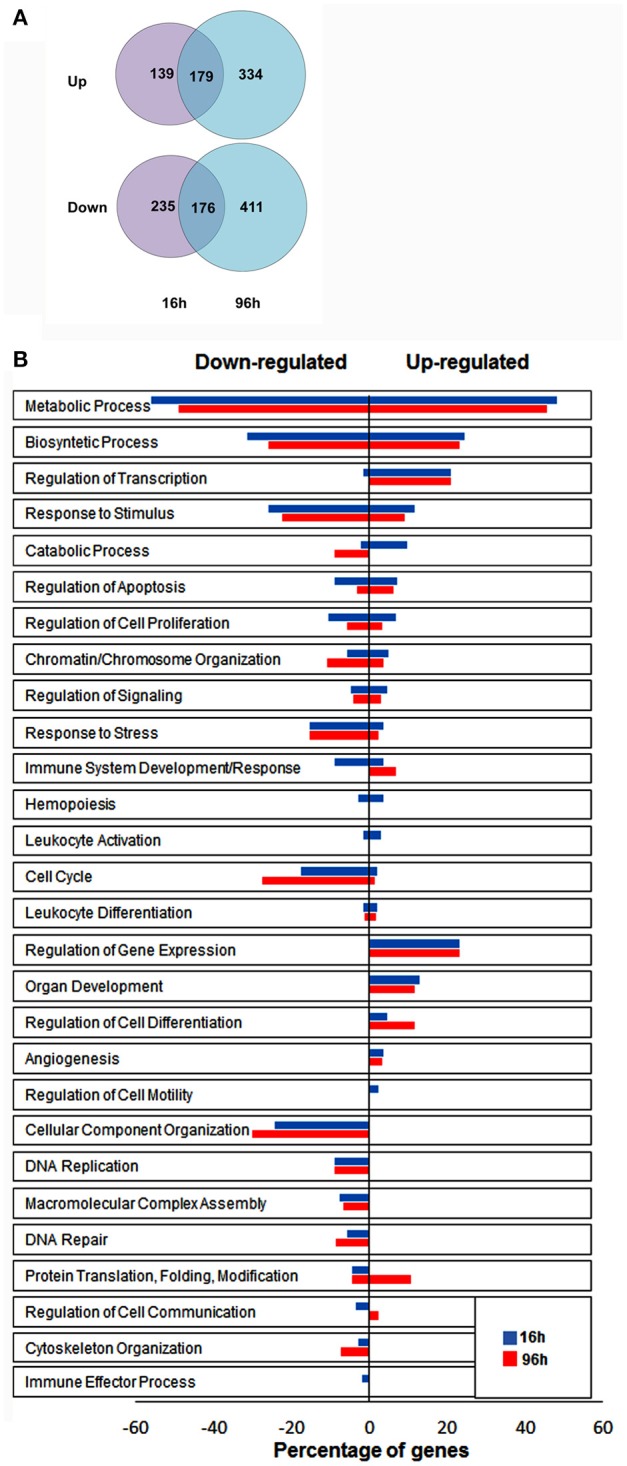
Identification of genes significantly modulated by hypoxia in NK cells by differential expression analysis. **(A)** Graphical representation of transcripts differentially expressed in hypoxic vs. normoxic NK cells. The gene expression profile of NK cells isolated from 3 different donors and exposed to hypoxia for 16 h (top) or 96 h (bottom) was analyzed by microarray analysis, as described in the section Materials and Methods. The Venn diagram depicts the number of transcripts exhibiting ≥2 fold up- or down-regulation in hypoxic vs. normoxic cells at the two time points. About 24% of differentially expressed transcripts are common to the 16 and 96 h transcriptomes. **(B)** Functional assessment of hypoxia-responsive genes by GO enrichment analysis. Unique genes showing at least 2-fold change in expression levels between Hy-NK and NK cells were clustered into different biological processes using the DAVID GO enrichment analysis. Based on this classification scheme, genes were placed in more than one biological process if more than one function of the encoded protein was established. The y-axis shows the GO terms. The x-axis shows the percentage of genes within each process relative to the total amount of genes belonging to that process: bars on the right of the y axis represent upregulated genes; bars on the left of the y axis represent downregulated genes. The blue columns represent genes modulated at 16 h whereas the red column represent genes modulated at 96 h.

To identify novel genes affected by hypoxia in NK cells, we performed differential expression analysis of microarray data. We filtered transcripts for a differential expression of at least 2-fold changes and a *p* ≤ 0.05. Using these selection criteria, we identified a total of 1,474 transcripts that were significantly modulated under hypoxic vs. normoxic conditions, with expression changes ranging from 106-fold upregulation to 25-fold downregulation (Table S1). The majority of differentially-expressed transcripts were identified as unique genes named in the GenBank™, whereas the remaining transcripts were either unnamed expressed sequence tags or hypothetical. As shown by the Venn diagram in Figure 2A, 355 transcripts were up- [179] or down-[176] regulated at both time points, whereas 374 (139 induced and 235 repressed) and 745 (334 induced and 411 repressed) transcripts were specifically modulated at 16 or 96 h, respectively. These results provide the first indication that Hy-NK cell signature varies with the duration of exposure to hypoxia.

To gain insights into the biological processes modulated by hypoxia, we carried out a Gene Ontology (GO) enrichment analysis on the lists of up- and down-regulated transcripts. We identified 28 biological processes containing a statistically significant enrichment of hypoxia-modulated genes (HMGs) (Figure 2B). Most processes were represented at both time points, although with variable HMG enrichment. Metabolism and biosynthesis resulted as the most enriched processes (in both up- and down-regulated genes), followed by response to stimulus. Additionally, Hy-NK cell transcriptional profile was related to regulation of apoptosis, and response to stress, but also to cell proliferation, signaling, and chromatine/chromosome organization. Certain processes, including regulation of gene transcription and expression, and cell differentiation, were selectively enriched in upregulated genes, whereas processes related to cell cycle, cellular component organization, and DNA replication and repair were exclusively enriched in downregulated genes at both time-points.

Importantly, different immune-related processes, including immune system development/response, hemopoiesis, leukocyte activation and differentiation, angiogenesis, regulation of cell motility and communication, and immune effector processes, were enriched in a statistically significant percentage of HMGs, suggesting regulatory effects of hypoxia on NK cell-mediated immune responses.

### Characterization of cytokine/chemokine and receptor gene modulation in Hy-NK cells

As the effect of hypoxia on the NK cell capability of killing target cells has been previously assessed (39), we focused our analysis on genes involved in immunoregulation and migration. The evaluation of immune-related gene clusters highlighted by GO analysis led to the identification of 43 HMGs coding for cytokines and chemokines, their receptors, and/or associated signaling molecules (Table 2). Some of these genes (26) were rapidly modulated by hypoxia (at 16 h time-point), while the remaining genes (17) were modulated after longer exposure (96 h).

**Table 2:** Relative expression of genes encoding cytokines/chemokines and their receptors in H-NK vs. NK cells^∧^.

**Ref Seq RNA**	**Gene symbol**	**Full Name**	**Main function(s) of gene product**	**FoldChange^§^**	Modulated in^*^					**References**
					**Monocytes**	**MDM**	**iDCs**	**mDCs**	**T cells**	
**16 h**										
**Up-regulated**										
NM_000597	IGFBP2	Insulin-like growth factor binding protein 2	Member of a regulatory network controlling cell proliferation, migration, and apoptosis; functions as a transporter of IGFs from the circulation into tissues; has growth stimulatory effect on tumor cells; contributes to T-cell activation and proliferation	71.7	/	/	/	/	/	
NM_00102539	VEGFA	Vascular endothelial growth factor A	Member of the PDGF/VEGF growth factor family; mitogen for endothelial cell (EC); plays a central role in driving angiogenesis and vasculogenesis via stimulation of EC survival, proliferation and migration and inhibition of apoptosis; promotes monocytic cell recruitment/activation	28.7	Up	Up	Up	Up	Up	(37, 45, 46, 63, 65–67, 69)
NM_001124	ADM	Adrenomedullin	Angiogenic peptide vasodilator that belongs to the calcitonin/calcitonin gene-related peptide (CGRP)/amylin peptide family; may function as a hormone in circulation control; acts as a multifunctional regulatory peptide	12.9	Up	Up	Up	Up	/	(45, 63, 66)
NM_00104008	SPP1/OPN	Secreted phosphoprotein (Osteopontin)	Pleiotropic cytokine/extracellular matrix phosphoprotein; plays a critical role in the activation of type I immunity and tumor growth, progression, and spread; endowed with EC adhesive capacity and chemotactic activity for EC, monocytes, and T lymphocytes	11.6	Up	Up	Up	Up	/	(37, 45, 66)
NM_000584	CXCL8	Chemokine (C-X-C motif) ligand 6 (IL-8)	Member of the a-chemokine family; functions as a neutrophil chemotactic and activating factor, potent angiogenic factor and major mediator of the inflammatory response	4.9	/	Up	Up	Up	Up	(37, 46, 63, 65–67)
NM_003467	CXCR4	Chemokine (C-X-C motif) receptor 4	Receptor of the stromal cell-derived factor-1; key regulator of cell motility and migration; critical for retention and homing of hematopoietic cells, including NK cells, in the BM; plays important roles in tumor cell metastatization in many types of cancers	3.4	Up	Up	Up	/	Up	(45, 46, 63, 65, 67, 68)
NM_003377	VEGFB	Vascular endothelial growth factor B	Member of the PDGF/VEGF growth factor family; ligand for VEGF receptor 1 and neuropilin-1; regulates the formation of blood vessels and is involved in EC physiology controlling uptake of fatty acids	3.2	/	/	/	/	/	
NM_003840	TNFRSF10D (TRAILR4)	Tumor necrosis factor receptor superfamily, member 10d	Member of the TNF-receptor superfamily; receptor of TNF-related apoptosis-inducing ligand (TRAIL); plays an inhibitory role on TRAIL-induced cell apoptosis	2.5	/	Up	/	Up	/	(37)
NM_000875	IGF1R	Insulin-like growth factor 1 receptor	High affinity receptor of insulin-like growth factor; functions as an anti-apoptotic agent by enhancing cell survival; mediates regulation of NK cell development and cytotoxicity by IGF-1	2.4	/	/	/	/	/	
NM_00113559	TGFB2	Transforming growth factor, beta 2	Member of the TGFB family of cytokines; interferes with antitumor immune responses; has suppressive effects of interleukin-2 dependent T-cell growth; responsible for the down-regulation of the activating immunoreceptor NKG2D expression in CD8(+) T and NK cells and suppression of their lytic functions; may act directly as a tumor progression factor	2.3	/	/	/	/	/	
NM_002415.1	MIF	Macrophage migration inhibitory factor	Pleiotropic cytokine with multiple effects in immunoregulation and inflammation; promoter of tumor cell proliferation, migration, metastasis, and tumor angiogenesis; suppressor of p53-mediated apoptosis and antitumor immunity; inhibitor of macrophage motility and NK cell cytotoxicity against tumor cells by down-regulating NKG2D receptor	2.1	Up	Up	Up	Up	Up	(37, 45, 46, 63, 65, 66)
**Down-regulated**										
NM_002309	LIF	Leukemia inhibitory factor (cholinergic differentiation factor)	Member of the IL6 family of cytokines; displays pleiotropic effects on various cell types and organs; involved in the induction of myeloid hematopoietic and neuronal cell differentiation; regulator of mesenchymal to epithelial conversion; plays a key anti-inflammatory role in cutaneous inflammation; promotes Treg development and adaptive immune tolerance; involved in immune tolerance at the maternal-fetal interface	0.21	/	/	/	/	/	
NM_003701.3	TNFSF11 (RANKL)	Tumor necrosis factor (ligand) superfamily, member 11	Member of the TNF cytokine family; functions as a key factor for osteoclast differentiation and activation; involved in the regulation of T cell immune response and DC survival; may have a role in the regulation of apoptosis; involved in oncogenesis, tumor progression, and metastatization	0.27	/	/	/	/	/	
NM_00115970	LTA (TNFSF1)	Lymphotoxin alpha (TNF superfamily, member 1)	Member of the TNF family, forms heterotrimers with lymphotoxin-beta; mediates inflammatory, immunostimulatory, and antiviral responses; involved in secondary lymphoid organ formation during development; plays a role in tumor apoptotic killing byNK cells	0.33	/	/	/	/	/	
NM_002341.1	LTB (TNFSF3)	Lymphotoxin beta (TNF superfamily, member 3)	Type II membrane protein of the TNF family; anchors lymphotoxin-alpha to the cell surface through heterotrimer formation; inducer of inflammatory responses; involved in development of lymphoid tissue; plays a role in tumor apoptotic killing by NK cells and in IL-12 production	0.34	/	/	/	/	/	
NM_002995	XCL1 (ATAC)	Chemokine (C motif) ligand 1 (Lymphotactin)	Member of the C-chemokine subfamily produced by T, NK, and NKT cells during infectious and inflammatory responses; functions in inflammatory and Th1-type immune responses; induces T cell migration and activation, DC-mediated cytotoxic activity; regulates thymic establishment of self-tolerance and generation of Treg	0.34	/	/	/	Up	/	(37)
NM_004195	TNFRSF18	Tumor necrosis factor receptor superfamily, member 18 (GITR)	Member of the TNF-receptor superfamily, binds TNFSF18; modulates T-lymphocyte survival in peripheral tissues, important for interactions between activated T-lymphocytes and ECs; participates in the development of autoimmune/inflammatory responses and graft-vs- host disease and potentiates response to infection and tumors through activation of effector T-cells, inhibition of regulatory T (Treg) cells, NK-cell co-activation, activation of macrophages, modulation of DC function	0.38	/	/	/	Up	/	(37)
NM_000619	IFNG	Interferon, gamma	Member of the type II interferon family; endowed with antiviral, immunoregulatory, anti-angiogenic, anti-proliferative, and pro-apoptotic properties; promotes tumor immunogenicity; potent activator of macrophages; critical for NK-mediated CTL activation and Th1 cell development	0.39	/	/	/	/	Down	(69)
NM_000594	TNFA (TNFSF2)	Tumor necrosis factor-alpha	Multifunctional proinflammatory cytokine that belongs to the TNF superfamily; involved in the regulation of several biological processes including immune cell proliferation, differentiation, migration, and activation, apoptosis, necrosis, lipid metabolism, and coagulation; implicated in autoimmune diseases, insulin resistance, and tumor immune surveillance	0.42	Up	Up	Down	/	Down	(45, 46, 63, 67)
NM_00100147	CCL3 (MIP-1a)	Chemokine (C-C motif) ligand 3	Member of the beta-chemokine subfamily; plays a role in inflammatory responses; induces monocyte, activated T cell, immature DC, and NK cell chemotaxis; inhibits HIV replication; plays a role in NK cell cytolytic and antiviral activity	0.43	/	Up	/	Up	Up	(37, 46, 63)
NM_172014	TNFSF14 (LIGHT)	Tumor necrosis factor (ligand) superfamily, member 14	Member of the TNF ligand family, binds to TNFRSF14 and LTβR on hematopoietic and stromal cells; functions as a costimulatory factor for lymphoid cells and as a deterrent to infection by herpesvirus; stimulates T cell proliferation; triggers tumor cell apoptosis; plays a critical role in NK activation/expansion and activated NK cell and DC priming of CD8+ T cells; increases effector cell priming, recruitment, and retention at tumor sites	0.43	Up	/	Up	/	Down	(45, 46, 65)
NM_000634	CXCR1	Chemokine (C-X-C motif) receptor 1	Member of the G-protein-coupled receptor family; high affinity receptor of CXCL8; mediates NK cell trafficking in the bone marrow (BM) under physiological conditions and recruitment to inflammatory sites	0.45	/	/	/	/	/	
NM_001295	CCR1	Chemokine (C-C motif) receptor 1	Member of the betachemokine receptor family, binds CCL3, CCL5, CCL7, and CCL23; mediates NK cell trafficking in the BM under homeostatic conditions and recruitment of BM-derived NK cells to inflammatory and tumor sites; increases NK cells cytolitic activity	0.49	Down	/	/	/	/	(45)
NM_000579	CCR5	Chemokine (C-C motif) receptor 5	Member of the beta-chemokine receptor family, binds CCL3, CCL4, CCL5, and CCL8; plays a role in NK cell proliferation and circulation under physiological conditions; mediates recruitment of BM-derived NK cells to sites of inflammation and tumors; enhances NK cell cytolitic activity; co-receptor for macrophage-tropic virus, including HIV	0.49	Down	Down	Up	Up	Up	(45, 46, 64, 66–68)
NM_001565	CXCL10 (IP-10)	Chemokine (C-X-C motif) ligand 10	Member of the alpha-chemokine subfamily; ligand for the receptor CXCR3; IFNg-inducible chemokine endowed with pleiotropic effects; inducer of monocytes, NK and T-cell migration; inhibitor of angiogenesis; modulator of adhesion molecule expression	0.50	/	/	Up	/	/	67
NM_001504	CXCR3	Chemokine (C-X-C motif) receptor 3	G protein-coupled receptor with selectivity for CXCL9, CXCL10, and CXCL11; induces integrin activation, cytoskeletal changes, and chemotactic migration; promotes NK cell trafficking in the BM and accumulation into tumors; enhances NK cell antitumor activity	0.50	/	/	/	/	/	
**96 h**										
**Up-regulated**										
NM_001124	ADM	Adrenomedullin	Angiogenic peptide vasodilator that belongs to the calcitonin/calcitonin gene-related peptide (CGRP)/amylin peptide family; may function as a hormone in circulation control; acts as a multifunctional regulatory peptide	9.2	Up	Up	Up	Up	/	(45, 63, 66)
NM_006944	SPP2	Secreted phosphoprotein 2, 24kDa	Secreted phosphoprotein member of the cystatin superfamily; NFkappaB-dependent gene with a pro-inflammatory role	7.4	/	/	/	/	/	
NM_00102539	VEGFA	Vascular endothelial growth factor A	Member of the PDGF/VEGF growth factor family; mitogen for endothelial cell (EC); plays a central role in driving angiogenesis and vasculogenesis via stimulation of EC survival, proliferation and migration and inhibition of apoptosis; promotes monocytic cell recruitment/activation	7.2	Up	Up	Up	Up	Up	(37, 45, 46, 63, 65–67)
NM_000584	CXCL8	Chemokine (C-X-C motif) ligand 6 (IL-8)	Member of the a-chemokine family; functions as a neutrophil chemotactic and activating factor, potent angiogenic factor and major mediator of the inflammatory response	6.4	/	Up	Up	Up	Up	(37, 46, 63, 65–67)
NM_004112	FGF11	Fibroblast growth factor 11	Member of the FGF family with mitogenic, cell survival, proangiogenic, and tumorigenic functions	6.0	/	/	/	/	/	
NM_000597	IGFBP2	Insulin-like growth factor binding protein 2	Member of a regulatory network controlling cell proliferation, migration, and apoptosis; functions as a transporter of IGFs from the circulation into tissues; has growth stimulatory effect on tumor cells; contributes to T-cell activation and proliferation	5.8	/	/	/	/	/	
NM_00101383	PTAFR	Platelet-activating factor receptor	Receptor for platelet-activating factor with pleiotropic functions; regulator of cell motility, smooth muscle contraction, leukocyte migration, and cytokine/chemokine production; implicated in allergy, asthma, septic shock, arterial thrombosis, and inflammatory processes; involved in immunosuppression	3.5	/	/	/	/	/	
NM_001616	ACVR2A	Activin A receptor, type IIA	Receptor of activins, which are members of the TGF-beta superfamily involved in several biological processes.	3.1	/	/	/	/	/	
NM_000875	IGF1R	Insulin-like growth factor 1 receptor	High affinity receptor of insulin-like growth factor; functions as an anti-apoptotic agent by enhancing cell survival; mediates IGF-1-dependent promotion of NK cell development and cytotoxicity	3.1	/	/	/	/	/	
NM_003840	TNFRSF10D	Tumor necrosis factor receptor superfamily, member 10 d (TRAILR4)	Member of the TNF-receptor superfamily; receptor of TRAIL; plays an inhibitory role in TRAIL-induced cell apoptosis	3.0	/	/	/	/	/	
NM_002185	IL7R	Interleukin 7 receptor	Receptor of interleukine 7; essential for T lymphocyte development, survival, and proliferation	2.8	/	/	/	/	/	
NM_003839	TNFRSF11A (RANK)	Tumor necrosis factor receptor superfamily, member 11a	Member of the TNF-receptor superfamily; can interact with various TNF-receptor associated factor (TRAF) family proteins, activator of the PI3K-Akt pathway; regulator of T/DC interaction; essential mediator of osteoclast differentiation and activation	2.6	/	/	Down	/	/	(65)
NM_016639	TNFRSF12A (TWEAKR)	Tumor necrosis factor receptor superfamily, member 12A	Member of the TNF-receptor superfamily; activator of the NF-kB and PI3K-Akt signaling pathways; mediator of inflammation and tissue remodeling; plays a role in the pathogenesis of inflammatory and systemic autoimmune diseases	2.5	/	/	Up	/	/	(65)
NM_00114531	CX_3_CR1	Chemokine (C-X3-C motif) receptor 1	Receptor for fractalkine/CX3CL1; plays a role in NK cell adhesion, egress from BM, and migration to sites of disease mediated by CX3CL1 and CCL26; involved in NK cell cytotoxicity against tumor cells expressing CX3CL1	2.4	up	/	Up	/	/	(45, 65)
NM_033135	PDGFD	Platelet derived growth factor D	Member of the PDGF family; potent pro-tumorigenic and angiogenic factor; stimulates matrix metalloprotease activities and monocyte/macrophage migration; contributes to epithelial-mesenchymal transition (EMT)	2.2	/	/	/	/	/	
NM_031950	FGFBP2	Fibroblast growth factor binding protein 2	Member of the FGF binding protein family selectively secreted by cytotoxic lymphocytes and involved in cytotoxic lymphocyte-mediated immunity	2.2	/	/	/	/	/	
**Down-regulated**										
NM_002309	LIF	Leukemia inhibitory factor (cholinergic differentiation factor)	Member of the IL6 family of cytokines; displays pleiotropic effects on various cell types and organs; involved in the induction of myeloid hematopoietic and neuronal cell differentiation; regulator of mesenchymal to epithelial conversion; plays a key anti-inflammatory role in cutaneous inflammation; promotes Treg development and adaptive immune tolerance; involved in immune tolerance at the maternal-fetal interface	0.17	/	/	/	/	/	
NM_153460	IL17RC	Interleukin 17 receptor C	Component of theIL-17R complex with IL-17RA; mediates the functions of both IL-17A and IL-17F proinflammatory cytokines; involved in host defense, innate immunity, tissue remodeling, and acute phase responses; implicated in the progression of inflammatory and autoimmune diseases; enhances IFNg secretion by NK cells	0.19	/	/	/	/	/	
NM_003701.3	TNFSF11 (RANKL)	Tumor necrosis factor (ligand) superfamily, member 11	Member of the TNF cytokine family; functions as a key factor for osteoclast differentiation and activation; involved in the regulation of T cell immune response and DC survival; may have a role in the regulation of apoptosis; involved in oncogenesis, tumor progression, and metastatization	0.21	/	/	/	/	/	
NM_003856	IL1RL1 (IL-33R)	interleukin 1 receptor-like 1	Member of the interleukin 1 receptor family; receptor for IL-33; promotes both Th1 and Th2 immune responses; activates IFNg production from NK cells	0.36	/	/	/	/	/	
NM_006417.4	IFI44	interferon-induced protein 44	Microtubule-associated protein; member of the family of type I interferon-inducible genes; mediator of interferon alpha/beta antiviral and antiproliferative activities	0.36						
NM_006332.4	IFI30	interferon, gamma-inducible protein 30	Gamma-interferon-inducible lysosomal thiol reductase; enhances antigen presentation of a subset of MHC class II-restricted epitopes from disulfide bond-containing antigens.	0.39						
NM_000579	CCR5	chemokine (C-C motif) receptor 5	Member of the beta-chemokine receptor family, binds CCL3, CCL4, CCL5, and CCL8; plays a role in NK cell proliferation and circulation under physiological conditions; mediates recruitment of BM-derived NK cells to sites of inflammation and tumors; enhances NK cell cytolitic activity; co-receptor for macrophage-tropic virus, including HIV	0.44	Down	Down	Up	Up	Up	(45, 46, 64, 66–68)
NM_003810	TNFSF10 (TRAIL)	Tumor necrosis factor (ligand) superfamily, member 10	Member of the TNF ligand family; binds to several members of TNF receptor superfamily; induces apoptosis in transformed and tumor cells triggering activation of MAPK8/JNK, caspase 8, and caspase 3; plays a role as an effector molecule in NK cell tumoricidal activity; implicated in immunosuppressive, immunoregulatory and immune-effector functions; plays a role in immune response to viral infections and tumor immune surveillance	0.49	Down	/	/	/	Down	(45, 46)

∧ *NK cells were cultured under normoxic (20% O2) and hypoxic (1% O2) conditions for 16 and 96 h, and gene expression profiling was then carried out independently by microarray analysis on the RNA purified from three independent preparations. Comparative analysis of gene expression differences between the two experimental conditions was conducted as described in the section Materials and Methods. A GeneBank accession number, a common gene symbol, a full name, a brief description of the gene product main functions, and the fold change value (≥2-fold increase, ≤ 0.5-fold decrease) are specified for each gene. Genes in each group are ordered by fold change. Those validated by qRT-PCR are underlined*.

§ *Results are expressed as ratio of fold differences between hypoxic and normoxic samples (mean of expression level of three experiments). Genes up/downmodulated by ≥2-fold are shown*.

* *Comparison of microarray results with data previously obtained in monocytes, monocyte-derived macrophages, immature and mature dendritic cells, and T cells exposed to hypoxia*.

Compared to their normoxic counterparts, Hy-NK cells showed increased expression of genes coding for molecules with a primary role in angiogenesis (VEGFA, VEGFB, ADM, SPP1, FGF11) and promotion of inflammation or lymphocye cytotoxic responses (SPP1, SPP2, VEGF, TNFRSF11A and 12A, IGFBP2, FGFBP2, PTAFR), but also in apoptosis inhibition (IGF1R, TNFRSF10D, 11A, and 12A), tumor progression (IGFBP2, SPP1, MIF, PDGFD, TGFβ2, FGF11), and immunosuppression (MIF and TGFβ2) (34, 37, 48, 50–55). On the other hand, hypoxia inhibited mRNAs coding for cytokines and/or receptors mainly involved in anti-tumor and anti-viral immune responses including IFNγ, IFI30, IFI44, IL-17RC, IL1RL1, and various components of the tumor necrosis factor (TNF) superfamily, such as TNFα, LTA, LTB, TNFSF10,11,14, and TNFRSF18 (55–63). The only exception was represented by the inhibition of mRNA coding for LIF, a pleiotropic factor involved in the regulation of inflammation.

The Hy-NK transcriptome was also characterized by the differential modulation of genes coding for chemokines and chemokine receptors (11, 15). Specifically, we observed hypoxia-dependent upregulation of the mRNA for CXCL8 (which also has proangiogenic properties) (11, 37, 52), and downregulation of mRNAs for CXCL10, CCL3, and XCL1 (9, 11, 52, 64). Regarding the chemokine receptors known to be important for NK cell migratory activity mRNAs coding for CXCR4 and CX_3_CR1 were selectively up-regulated, whereas those coding for CXCR1, CCR1, CCR5, and CXCR3 were downregulated.

**Figure 3:**
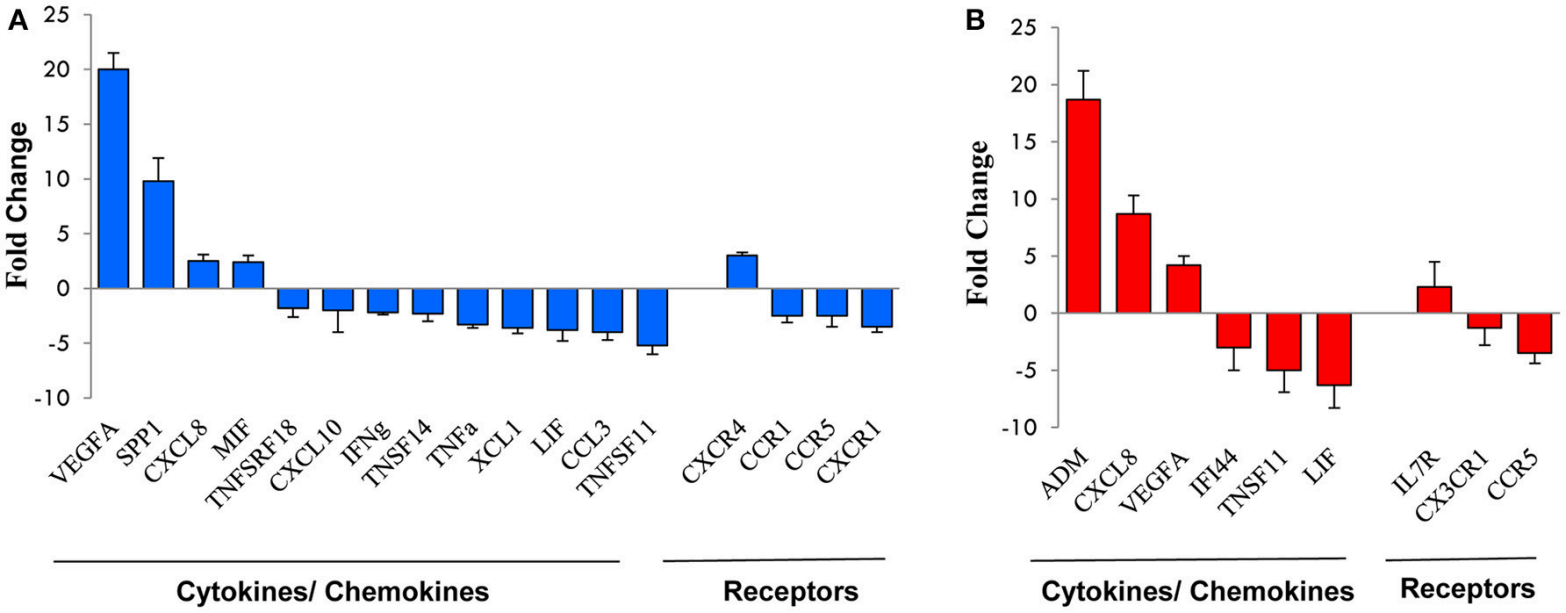
qRT-PCR validation of genes selected from the microarray profile. Total RNA from the NK cell preparations analyzed by microarray was subjected to qRT-PCR for the expression of a subset of genes randomly selected from those up- or down-modulated at 16 h **(A)** or 96 h **(B)**. Expression changes were evaluated in relation to the values obtained for three reference genes, as detailed in the section Materials and Methods. Results are expressed as fold-changes (Hy-NK relative to NK cells) and represent the mean of three determinations for each transcript. Positive values indicate that the mRNA levels of a specific gene was up-regulated, whereas negative values indicate that the transcript was down-regulated. Genes are ordered by fold-change within each group.

Microarray results were validated by qRT-PCR analysis of a subset of HMGs (17 belonging to the 16 h transcriptome and 9 to the 96 h transcriptome). As shown in Figure 3 and Table 2 there was almost full concordance between qRT-PCR and Affymetrix data with respect to the direction of the expression changes, with the only exception of CX_3_CR1 whose upregulation by hypoxia was not confirmed by qRT-PCR. For about half of validated genes, the extent of modulation was also comparable to that shown by microarray data, whereas it was higher for nine genes and lower for three genes. Such discrepancies, however, are consistent with previous findings showing that these techniques can often differently estimate the extent of gene regulation (42, 48).

A literature survey indicated that some of the HMGs in NK cells were targeted by hypoxia in different cell types including T lymphocytes (49), primary monocytes (48), monocyte-derived macrophages (MDMs) (38, 65, 66), immature (i)DCs (67–70), and mature (m)DCs (37, 68) (Table 2). In particular, MIF- and VEGFA-coding genes were upregulated by hypoxia in all the immune cell types analyzed, ADM and SPP1 were increased in the innate immune cells, while CXCL8 and CXCR4 upregulation was reported in T cells and in some mononuclear phagocyte populations. Other genes were variably modulated depending on the analyzed cell type. To our knowledge, a consistent part of the cytokine/chemokine- and receptor-coding genes identified in Hy-NK cells have not been reported to be modulated by hypoxia in other immune cell populations analyzed.

Taken together, these data indicate that hypoxia can induce a specific cytokine/chemokine and receptor gene signature in NK cells with possible functional consequences. To assess this possibility, we proceeded with the overall evaluation of the effects of hypoxia on NK cell immune-regulatory and migratory functions.

### Effects of hypoxia on NK cell-mediated release of chemokines and cytokines

**Figure 4:**
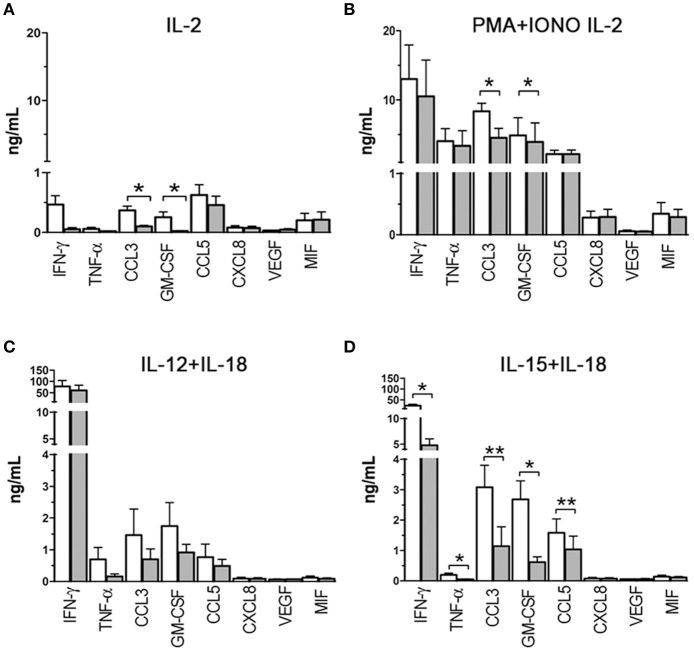
Cytokine/chemokine release capability of NK cells exposed to hypoxia or normoxia. Cell-free supernatants from NK cells cultured in the presence of IL-2 **(A)**, IL-2 and PMA+ionomycin **(B)**, IL-12+IL-18 **(C)**, IL-15+IL-18 **(D)** under normoxic (white columns) or hypoxic (gray columns) conditions were analyzed for 8 cytokine/chemokine content using the MAGPIX® System. Results are expressed as ng/ml and are the mean + SEM of 5 independent experiments. In **(A,C,D)**, the cells were cultured for 20 h in the presence of the indicated stimulating cytokines, in **(B)**, the cells were cultured for 26 h in the presence of IL-2 with the addition of PMA+ionomycin in the latter 6 h. * *p* < 0.05, ** *p* < 0.01.

To assess the effect of hypoxia on cytokine/chemokine release, PB-NK cells were freshly isolated from additional donors and cultured in the presence of IL-2 under hypoxic or normoxic conditions. After 20 h, supernatants were collected and analyzed by multiplex immunoassay for the content of IFNγ, TNFα, CCL3, GM-CSF, CCL5, CXCL8, VEGF, and MIF (Figure 4), namely those factors that are typically released by NK cells (6, 12) and/or that were shown to be transcriptionally affected in microarray analysis (Table 2). As shown in Figure 4A, upon exposure to IL-2 NK cells released low levels of different factors, including IFNγ, CCL3, GM-CSF, CCL5, and MIF. Under hypoxic conditions, release of IFNγ, CCL3, GM-CSF, and CCL5 was decreased (although differences reached statistical significance only for CCL3 and GM-CSF). Since IL-2 has typically limited direct effects on cytokine release while it primes NK cells to respond to other stimuli, we assessed the effect of hypoxia on NK cells cultured for 20 h with IL-2 and stimulated for further 6 h with PMA + Ionomycin (PMA+IONO). As shown in Figure 4B, an inhibitory effect on CCL3 and (slightly) on GM-CSF release was induced by hypoxia also on PMA+IONO-stimulated NK cells.

We next assessed the effect of hypoxia on NK cells cultured with other classical NK-activating stimuli, such as the monokines, IL-12, IL-15, and IL-18. In particular, we set monokine combinations known to potently stimulate NK cell cytokine secretion (i.e., IL-12+IL-18, and IL-15+IL-18). As shown in Figure 4C, NK cells cultured in the presence of IL-12 + IL-18 for 20 h released into the culture supernatant very high amounts of IFNγ and moderate to low amounts of CCL3, GM-CSF, TNFα, and CCL5. Hypoxia did not modify significantly NK cell ability to release cytokines in response to IL-12+IL-18, although a trend toward inhibition was observed for CCL3, GM-CSF, and TNFα release. Compared to IL-12+IL-18, IL-15+IL-18 stimulation induced lower release of IFNγ and TNFα and higher secreted levels of CCL3, GM-CSF, and CCL5 under normoxic conditions (Figure 4D). Exposure to hypoxic conditions resulted in the significant inhibition of IFNγ, TNFα, CCL3, GM-CSF, and CCL5 release.

We conclude from these data that hypoxia can differently affect cytokine/chemokine release depending on the type of stimulus. Specifically, it can modulate the release of only a few cytokines/chemokines in NK cells cultured in the presence of IL-2 or IL-2+PMA+IONO, exert a more general inhibition of cytokine/chemokine secretion on NK cells exposed to IL-15+IL-18, while it has no significant effects on NK cells exposed to IL-12+IL-18.

### Effect of hypoxia on chemokine receptor expression

**Figure 5:**
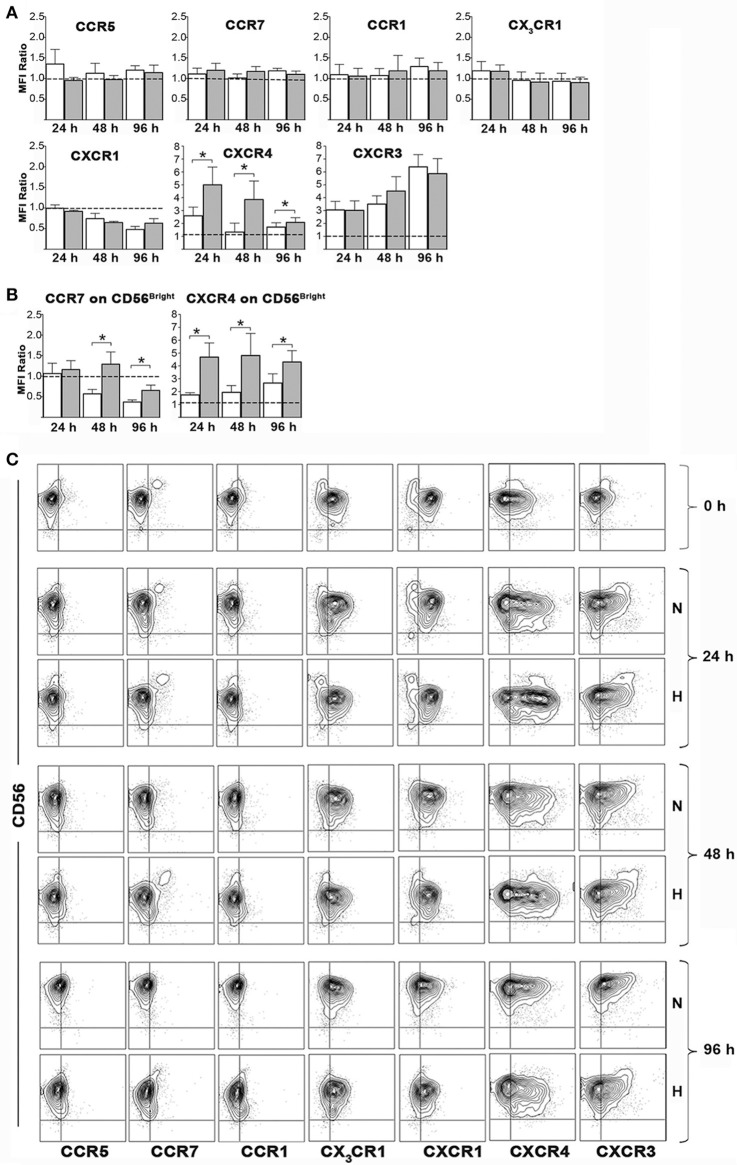
Effects of hypoxia on NK cell chemokine receptor expression. Freshly isolated PB-NK cells were cultured in the presence of IL-2 for 24, 48, or 96 h under normoxic or hypoxic conditions and then analyzed by flow cytometry for surface expression of the indicated receptors. **(A,B)** The ratio between the MFI observed at the indicated culture time points and that at t0 (i.e., on freshly isolated cells) is reported for each receptor. Horizontal dotted lines indicate no changes. White and gray bars are referred to NK cells cultured under normoxic or hypoxic conditions, respectively. Data are the mean + SEM of 5 independent experiments. In **(B)** data on CD56^bright^ gated cells are reported for CCR7 and CXCR4 expression. **(C)** FACS profiles of a representative donor are shown. * *p* < 0.05.

We next assessed whether hypoxia could modulate chemokine receptor expression on NK cell surface. To this end, freshly isolated PB-NK cells were cultured in the presence of IL-2 under hypoxic or normoxic conditions and analyzed by flow cytometry for the expression of CCR5, CCR7, CCR1, CX_3_CR1, CXCR1, CXCR4, and CXCR3 immediately after isolation or after 24, 48, or 96 h of culture. Upon exposure to IL-2 (under normoxic conditions), NK cells progressively down-regulated expression of CXCR1, up-regulated that of CXCR3 and (transiently) that of CXCR4, while they minimally modified the expression of CCR5, CCR1, and CX_3_CR1. Hypoxia further significantly increased the up-regulation of CXCR4 expression, slightly decreased CCR5 expression, while it did not substantially modify the expression trend of CCR1, CX_3_CR1, CXCR1, and CXCR3 (Figures 5A,C).

The analysis of CCR7 on the whole PB-NK cell population didn't give meaningful data, as CCR7 expression is generally confined to the small fraction of CD56^bright^ cells (12, 17) (Figures 5A,C). On the other hand, CD56^bright^ NK cells showed progressive decrease of CCR7 expression during culture with IL-2. Hypoxia significantly reversed such effect sustaining CCR7 expression on CD56^bright^ cells (Figures 5B,C). Remarkably, a careful analysis of such NK cell subset revealed that also CXCR4 expression could be sustained by hypoxia in CD56^bright^ cells (Figures 5B,C).

### Effects of hypoxia on NK cell chemotaxis

Experiments were then carried out to assess whether hypoxia-induced changes of CCR7 and CXCR4 expression could affect specific chemotactic activity of NK cells. To this end, NK cells cultured under normoxic or hypoxic conditions for 24, 48, and 96 h were analyzed in classical migration assays using CCL19, CCL21 (CCR7 ligands) and CXCL12 (CXCR4 ligand) as chemoattractants.

**Figure 6:**
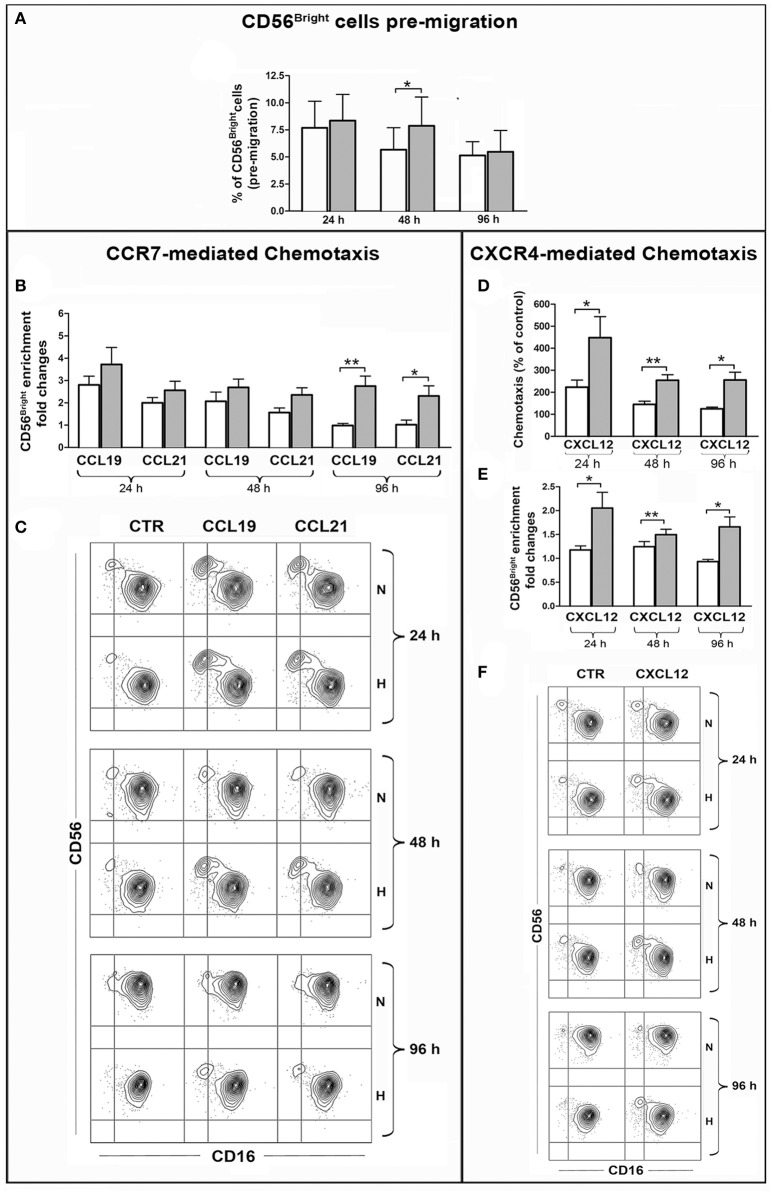
Effect of hypoxia on chemotaxis of PB-NK cells and their CD56^bright^/CD56^dim^ subset to CXCL12, CCL19, CCL21. PB-NK cells were cultured in the presence of IL-2 for 24, 48, or 96 h under normoxic or hypoxic conditions and analyzed by FACS for the combined expression of CD56 and CD16 markers in order to assess the percentage of the CD56^bright^CD16^dim/neg^ cells before migration **(A)**. Cells were then assessed for chemotaxis to the indicated chemokines under normoxic conditions for 2 h. Migrated cells were collected from the lower migration chamber compartments and counted or analyzed by FACS for the combined expression of CD56 and CD16 markers. The specific chemotactic response of CD56^bright^CD16^dim/neg^ NK cells to CCL19, CCL21 **(B)**, and CXCL12 **(E)** was assessed as enrichment of this cell subset within migrated cells. The enrichment was calculated as fold increase of the CD56^bright^CD16^dim/neg^ cell percentage within cells migrated to specific chemokines as compared to the CD56^bright^CD16^dim/neg^ cell percentage within spontaneously migrated cells (see section Materials and Methods for details). **(C,F)** Representative experiments showing the enrichment of CD56^bright^CD16^dim/neg^ NK cells within cells migrated in response to CCL19, CCL21 **(C)** or CXCL12 **(F)** as compared to cells that spontaneously migrated in the lower compartment in the absence of stimuli (CTR). **(D)** Specific chemotactic response to CXCL12 of the whole PB-NK cell population cultured under normoxic or hypoxic conditions. In **(B,D,E)** white and gray bars indicate data from NK cells cultured under normoxic or hypoxic conditions, respectively and represent the mean ± SEM of 6 independent experiments. * *p* < 0.05, ** *p* < 0.01.

Given the peculiar distribution of CCR7 within the PB NK cells, we analyzed whether CCL19 or CCL21 could induce the preferential migration of the CD56^bright^CD16^dim/neg^ cell subset, resulting in the enrichment of such population within migrated cells. Before performing this analysis, we evaluated by FACS whether the percentage of CD56^bright^CD16^dim/neg^ cells could be modified over time under hypoxic or normoxic culture conditions. As shown in Figure 6A the percentage of CD56^bright^ cells slightly decreased during culture under normoxic conditions, while hypoxia preserved such a population at the 24 and 48 h time points. This observation suggests that hypoxia could contribute to increase the absolute number of migrated CD56^bright^ cells by preserving them over time. In order to selectively evaluate the effect of hypoxia on specific chemotactic properties of the cells, the chemotactic response to chemokines was calculated as ratio of the CD56^bright^CD16^dim/neg^ cell percentages within cells migrated to specific chemokines or spontaneously. Among NK cells that have been cultured under normoxic conditions for 24 h, CD56^bright^ cells were able to migrate in response to both chemokines and enrich the population of migrated cells. However, this ability progressively disappeared at later culture time points. By contrast, Hy-NK cells gave rise to higher enrichment of CD56^bright^ cells within cells that migrated in response to CCL19/21 and maintained this capability over time (Figures 6B,C).

The analysis of CXCR4-dependent chemotaxis indicated that, overall, NK cells exposed to hypoxia were responsive to CXCL12 more than NK cells cultured under normoxic conditions (Figure 6D). Remarkably, this difference was more pronounced when considering the CD56^bright^ cell subset. Indeed, “hypoxic” (but not “normoxic”) NK cells gave rise to enrichment of the CD56^bright^ cells within cells migrated to CXCL12 (Figures 6E,F). These results were in line with the observation that under hypoxia all CD56^bright^ cells expressed CXCR4 at high levels while CD56^dim^ NK cells included a variable fraction of CXCR4neg cells (Figure 5C).

Overall, these data demonstrate that hypoxia can sustain CXCR4- and CCR7-dependent chemotactic response of NK cells to specific chemokines, such as CXCL12, CCL19, or CCL21, and favor the recruitment of CD56^bright^ cells, suggesting that a hypoxic environment may influence the extent and the nature of the NK cell infiltrate in different types of tumors.

## Discussion

In the present study we analyze the global effects of hypoxia on NK cells, which are among the most potent immune effectors available to the host for the control of tumor development and progression (1, 2, 6). We first provide the transcriptional overview of the response of IL-2-primed NK cells to short-term (16 h) and prolonged (96 h) hypoxia, demonstrating that Hy-NK cells are functionally reprogrammed through the differential expression of a large number of genes implicated in various aspects of NK cell biology, including immunoregulation and migration. Then, we document hypoxia influence on the chemotactic properties of specific NK cell subsets and on NK cell ability to release cytokines and chemokines, providing important clues on the effective role of the O_2_ tension in determining the composition and the function of the NK cell infiltrate in tumor lesions.

So far, one transcriptional study describing how hypoxia could influence the cytokine-mediated activation of NK cells has been done for IL-15, while no data were available on IL-2, although this factor represents the most known and studied priming cytokine for NK cells. As assessed by GSEA, gene expression changes observed upon NK cell exposure to 1% O_2_ conditions follow a consensus hypoxia transcriptional profile. Several hypoxia-related and HIF-1α/HIF-2α target gene sets defined in previous studies are, in fact, significantly enriched in both the 16 and the 96 h Hy-NK transcriptomes. Moreover, we find enrichment of genes involved in glycolysis, gluconeogenesis, glucose transport, non-glycolytic metabolism, and ion transport, which is a common feature of hypoxic cells of different type, origin, and functional state being essential to compensate for the inhibition of oxidative metabolism and the malfunctioning of O_2_-dependent enzymes occurring under conditions of reduced oxygenation (32, 34, 47–49).

GO clustering of HMGs in NK cells indicates that hypoxia can modulate several biological processes and suggests that NK cells reaching hypoxic tumor areas may deeply change their mode to respond to stimuli or exert their functions. Processes related to metabolism and biosynthesis, response to stimuli, regulation of apoptosis and response to stress, cell proliferation, and signaling appear to be all affected by hypoxia suggesting that NK cells may modulate a wide range of functions in a hypoxic environment. In particular, the coordinated enrichment of down- or up-regulated genes in specific processes, such as cell cycle, DNA replication and repair, cellular component organization, regulation of gene transcription and expression, indicates that NK cells can moderate their biosynthetic and proliferative capabilities in response to decreased O_2_ tension.

Noteworthily, among HMGs we identified a significant cluster of immune-related genes, 43 of which coding for cytokines, chemokines, and their receptors. Several of these cytokine/chemokine-coding genes have not been previously reported to be affected by hypoxia in NK cells, although some of them are known from the literature to be modulated in other immune cells either exposed to short-term hypoxia (typically 8–24 h) (48, 49, 65, 66, 70, 71) or generated under conditions of long-term hypoxia (37, 38, 47, 67–69). On the other hand, some genes appear to be modulated uniquely in NK cells, as they have never been characterized in terms of responsiveness to hypoxia in other immune cells. These findings indicate that hypoxia regulates the expression of genes coding for cytokines/chemokines on different immune cell populations, but it can also activate a distinct transcriptional profile in NK cells.

The data on immune-related HMGs give some hints on how NK cells may be functionally skewed in a hypoxic microenvironment. The early downregulation of genes coding for IFNγ and for several members of the TNF family, such as TNFα, LTA, LTB, TNFSF14, TNFSF10, and TNFSF11, is of particular interest given the role of these molecules in triggering tumor immunogenicity, decreasing tumor proliferation and angiogenesis, and favoring apoptotic tumor cell killing. Likewise, the downregulation of genes coding for TNFRSF18 and IL1RL1 may be crucial, as these molecules act by enhancing IFNγ secretion and potentiating NK cell expansion and response to tumors (62, 63). Consistently, hypoxia also induces the up-regulation of genes coding for important proangiogenic, protumorigenic, prometastatic, and/or immune suppressive factors, namely VEGFA,B, SPP1,2, CXCL8, MIF, TGFβ2, and PDGFD (11, 34, 37, 48, 50–54). Finally, it is remarkable the modulation of genes coding for chemokines and chemokine receptors.

Collectively, our gene expression analysis suggests a major effect of hypoxia on the immunomodulatory functions and the chemotactic properties of NK cells. These aspects are not trivial, as the nature and the function of the NK cell infiltrate at the tumor site, as well as the tissue distribution of specific NK cell subsets, can influence the prognosis of different tumor types (11, 12, 28, 29). For this reason we decided to investigate in detail the effect of hypoxia on these specific functions of NK cells: cytokine/chemokine release and migration to specific stimuli.

Multiplex ELISA analysis of NK cell culture supernatants partly confirmed the indications obtained by the gene chip analysis, showing that, indeed, hypoxia can limit the ability of NK cells to release different factors involved in the host response to the tumor, such as IFNγ, TNFα, GM-CSF, CCL3, and CCL5. These factors are endowed with antitumor activity and/or can induce recruitment, differentiation, proliferation, and activation of APCs, Th1 lymphocytes, and NK cells (9, 11, 72, 73). Hypoxia appears to variably affect cytokine release, depending on the type of NK cell stimulation. Indeed, its inhibitory effect is particularly evident on NK cells exposed to the monokine combination, IL-15 + IL-18, while it does not reach statistical significance in case of IL-12 + IL18 stimulation. We couldn't find any transcriptional modulation of genes coding for IL-12, IL-15, IL-18, or IL-2 receptor subunits, suggesting that the differential effect of hypoxia on the various stimuli may involve other mechanisms such as the interference with specific signaling pathways.

That hypoxia could differentially modulate NK cells under different monokine stimuli is important both because stimulatory monokines can be released by immune cells in inflamed tissues and also in view of the recent lines of research aimed at the definition of effective monokine combinations in NK-based immunotherapy (2, 17, 74). Notably, the hypoxia-related factors CXCL8, VEGF, and MIF were not (or poorly) released by both “normoxic” and “hypoxic” NK cells, although they were induced by hypoxia at the mRNA level. This discrepancy suggests that target-specific translational regulation (49, 67) can shape NK cell response to hypoxia, giving rise to unique functional profiles. The fact that hypoxia fails to induce CXCL8, VEGF, and MIF secretion by NK cells has also been reported in a recent study by Velasquez et al. (41). In that study, however, in contrast to our present data, exposure to hypoxia could induce little, but significant, secretion of CCL3, CCL4, and CCL5. The discrepancy between our and their results may probably be ascribed to the different experimental protocols used, in particular with regard to the priming cytokines (IL-2, IL-12+IL-18, or IL-15+IL-18 in our study vs. IL-15 alone in that of Velasquez) and the time and duration of priming (the whole 20 h culture period in our study vs. the final 6 h culture in the Velasquez study).

Evaluation of chemokine receptor surface expression reveals that hypoxia can significantly increase the expression of CXCR4 receptor on a large fraction of PB-NK cells, suggesting that changes in the levels of O_2_ tension within tissues may significantly influence NK cell trafficking (11). The CXCL12-CXCR4 axis represents one of the mechanisms responsible for tumor spread, driven by pro-metastatic CXCR4+ tumor cells. In addition, CXCL12 expressed by Tumor Associated Fibroblasts (TAF) and tumor cells has been demonstrated to play an important role in favoring tumor growth and progression in primary lesions. Thus, sustained CXCR4 expression in NK cells may be important for reaching and infiltrating certain metastatic niches (for example in the bones) and also primary tumors. In this context, in a model of NKp46-targeted HIF1α KO mice, it has been recently shown that NK cells can reach hypoxic tumor tissues, influence angiogenesis, tumor growth, and metastasis spread in a HIF1α-dependent fashion (75). Remarkably, our data indicate that, in humans, hypoxia can differently affect two functionally distinct NK cell subsets. We observed that hypoxia-induced CXCR4 up-regulation involved the whole CD56^bright^ NK cell population, while it affected only a fraction (even if large) of CD56^dim^ cells. Accordingly, hypoxic NK cells that migrated to CXCL12 showed an enrichment of such CD56^bright^ cell subset. Along this line, hypoxia also increased CCR7 expression on CD56^bright^ cells, enhancing their selective migration in response to CCL19 and CCL21. The CCL19/21-CCR7 axis drives metastatic spread to Lymph Nodes but also promotes homing of specific leukocyte subsets. In addition, the CCL21-CCR7 interaction may be effective at the tumor site (76, 77). Overall, our data suggest that hypoxia can intervene in the recruitment of specific NK cell subsets at the site of both primary tumor and metastasis and offer new hints to explain the relative high frequencies of poorly cytotoxic CD56^bright^ cells observed within the NK cell infiltrate of several tumors (29–31). Of course these findings, although suggestive, must be considered within the rich network of factors that regulates lymphocyte trafficking in different tumor sites. As an example, it has been recently described the complex correlation between the chemokine receptor pattern of different T lymphocyte subsets and the control of metastasis in specific sites (78). Also NK cells can respond to multiple chemokines that can be variably released in different tumor sites. In this context, it is worth-noting that NK cells can amplify their recruitment at the tumor site by killing tumor cells and inducing release of chemiotactic HMGB1 (79).

Various escape mechanisms induced by the tumor hypoxic environment have been documented in the last years, most relying on the suppression of different immune cell types, others involving the editing of the tumor cell targets or the tumor microenvironment (23, 33, 38, 47, 80–85). However, only few studies were focused on NK cells. This report represents the first comprehensive transcriptome analysis of human Hy-NK cells, which defines a wide array of hypoxia-modulated immunological genes. Remarkably, our study also describes how hypoxia can influence the type and function of NK cells reaching hypoxic tissues, thus providing new elements useful to design improved NK cell-based immunotherapeutic strategies.

## Ethics statement

This study was carried out following approved operational procedures of the Ethics Committee of the IRCCS Ospedale Policlinico San Martino (IOH78). Written informed consent was obtained from all the donors in adherence with the Declaration of Helsinki.

## Author contributions

MP and FR conducted the experiments, assembled and analyzed data, and participated in MS writing. DC provided statistical analysis of microarray data and performed GSEA analysis. CM, MB, and FB provided experimental support and helped in the analysis of data. AE revised the manuscript. LV, GP, LM, and MM revised the manuscript and provided financial support. MV and MCB conceived the study, designed experiments, interpreted the data, wrote the manuscript, and provided financial support.

### Conflict of interest statement

The authors declare that the research was conducted in the absence of any commercial or financial relationships that could be construed as a potential conflict of interest.

## Abbreviations

PB: peripheral blood
pO_2_: low oxygen tension
GSEA: Gene Set Enrichment Analysis
qRT-PCR: Real time PCR
Hy-NK: hypoxic NK cells
HIF: hypoxia-inducible factor
NES: normalized enrichment score
FDR *q*-val: false discovery rate *q*-values
NOM *p*-val: nominal *p*-values
LEA: Leading Edge Analysis
GO: gene ontology
HMGs: hypoxia-modulated genes
MDMs: monocyte-derived macrophages
iDCs: immature DCs
mDCs: mature DCs
IONO: ionomycin.
