# Supplementary material for: Hypoxia Modifies the Transcriptome of Human NK Cells, Modulates Their Immunoregulatory Profile, and Influences NK Cell Subset Migration
Source: Front Immunol. 2018 Oct 16;9:2358. doi: 10.3389/fimmu.2018.02358 (PMC6232835; doi:10.3389/fimmu.2018.02358)
Supplement: Supplementary file 2 [file Data_Sheet_1.PDF]

Parodi M./Raggi F. et al.
SUPPLEMENTARY FIGURE

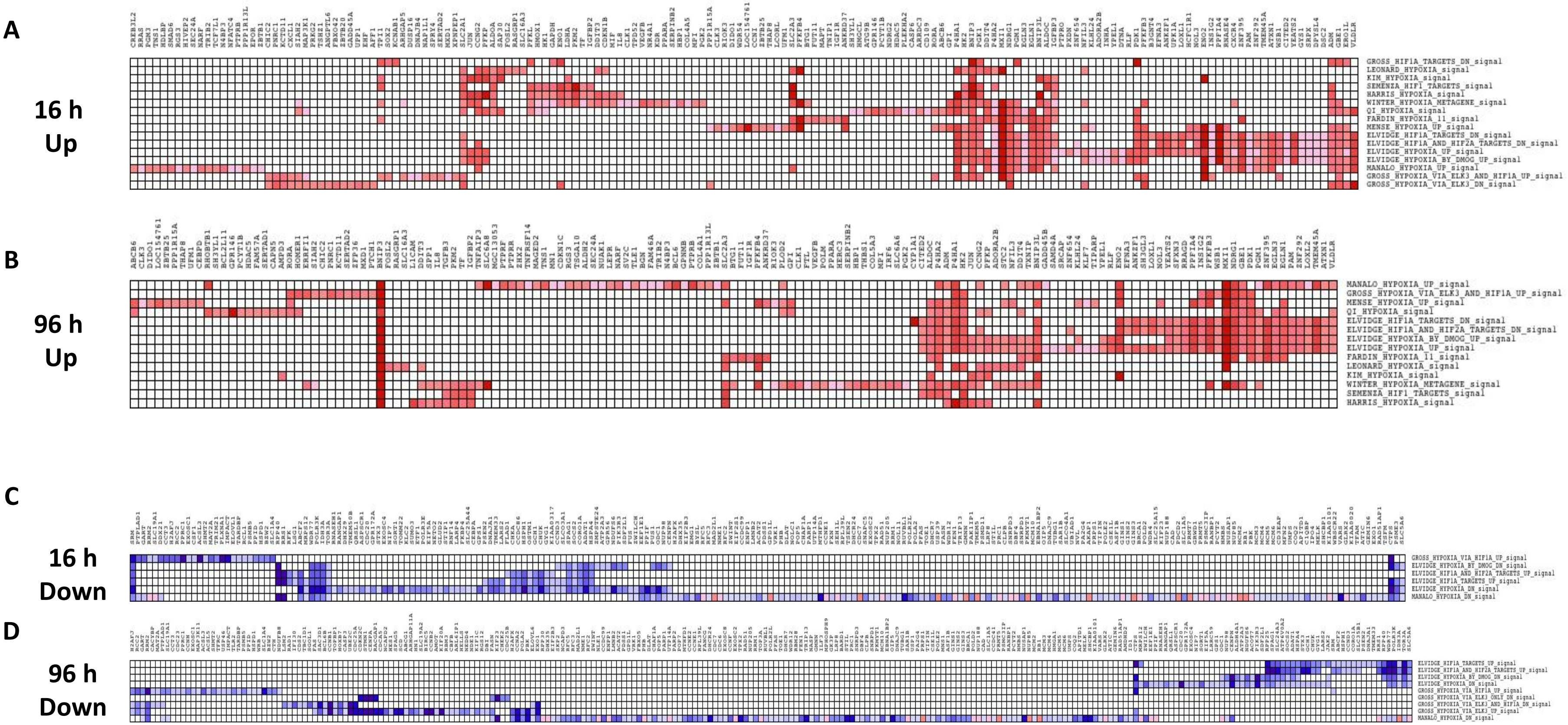

Figure S1. Heatmaps linking genes and hypoxia. Four heatmaps linking genes and hypoxia are shown. Each heatmap was generated by LEA of enriched gene sets identified by GSEA in the transcriptomes of NK cells exposed to hypoxia for 16h or 96h. (A-B) LEA heatmaps of gene sets specifically enriched in the up-regulated Hy-NK transcriptomes. (C-D) LEA heatmaps of gene sets specifically enriched in the down-regulated Hy-NK transcriptomes. Expression values are represented by a color scale with *red* and *pink* indicating genes respectively highly and moderately upregulated while *dark blue* and *light blue* indicating genes respectively highly and moderately downregulated in Hy-NK compared to NK cell trascriptomes.

Figure S1
